# General surgery and COVID-19: review of practical recommendations in the first pandemic phase

**DOI:** 10.1007/s00595-020-02086-4

**Published:** 2020-07-27

**Authors:** Vittorio Bresadola, Carlo Biddau, Alessandro Puggioni, Alessandro Tel, Massimo Robiony, Jonathan Hodgkinson, Cosimo Alex Leo

**Affiliations:** 1 General Surgery Department and Simulation Center, Academic Hospital of Udine, Department of Medicine, University of Udine, Udine, Italy; 2 Maxillofacial Surgery Department, Academic Hospital of Udine, Department of Medicine, University of Udine, Udine, Italy; 3 Department of General and Emergency Surgery, Northwick Park and St Mark’s Hospital, London North West University Healthcare NHS Trust, Harrow, UK; 4 Department of Surgery & Cancer, Imperial College London, London, UK

**Keywords:** SARS-CoV-2, COVID-19, Surgery, Management, Recommendations

## Abstract

**Background:**

In March, 2020, the World Health Organization declared COVID-19 a pandemic. The absence of previous knowledge of COVID-19 has made decision-making difficult for all in health care, including surgical departments. We reviewed the management recommendations for surgical activity and changes to surgical practice, identifying concordances and discrepancies, based on the literature published in the early phase of the pandemic.

**Method:**

We searched the electronic datasets, PubMed Database, Google, and Google Scholar, using the keywords “SARS-CoV-2”, “COVID-19”, “surgery”, “recommendations”, “guideline”, and “triage”. The search was limited to the first 2 months after the pandemic began and was closed on May 6, 2020.

**Results:**

Twenty papers were included in the analysis and their recommendations are divided into the following categories: 1. general aspects, such as maintaining the safety of health personnel and indications for surgery. 2. The preoperative phase, with recommendations about activating different care pathways for COVID-19 positive patients. 3. The operative phase, with recommendations about activating safety measures for aerosol-generating procedures. 4. The postoperative phase, with recommendations for managing operating theatres and patient transfers.

**Conclusion:**

The recommendations proposed in the revised documents are considered good practices aimed at keeping patients and healthcare professionals safe. However, these recommendations must be contextualized in each individual hospital.

## The scenario

On December 31, 2019, Chinese health authorities reported an outbreak of pneumonia of unknown etiology in Wuhan City, Hubei Province, China [1,2]. On January 9, 2020, the China Centre for Disease Control identified the causative agent as a new coronavirus called 2019-nCOV (officially SARS-CoV-2) [3,4]. On February 11, 2020, the World Health Organization (WHO) named the respiratory disease related to infection “COVID-19” (Corona Virus Disease) [5]. On March 11, 2020, the WHO declared the COVID-19 outbreak a pandemic [6].

In Italy, the first western country massively affected by the epidemic, the initial cases were identified in two Chinese tourists and confirmed by the Superior Institute of Health as COVID-19 on January 30, 2020. On February 21, 2020, the first indigenous case was identified in Lombardia in northern Italy [7]. Following rapid spread of the epidemic, the Council of Ministers launched a series of decree-laws with increasingly restrictive containment measures that defined a framework of complete lockdown on March 22, 2020 [8]. Unlike in emergencies with known dynamics, the sudden presentation of the pandemic, the lack of prior specific knowledge of the COVID-19 infection, the non-existence of literature on this topic and the lack of evidence-based medicine (EBM) guidelines have made it extremely difficult to plan responses in the first phase of the pandemic. This has also made the decision-making phase challenging in all areas of our society. Even in the surgical arena, important choices have had to be implemented quickly, often based on day-by-day experience.

In this initial pandemic period, the planning of surgical activity faced several challenges. These included making the necessary resources available for treating COVID-19 positive patients, such as ventilators, intensive care beds, and staff; ensuring the safety of both patients and health care workers with respect to the risk of COVID-19 infection, through the identification of infected subjects, the use of Personal Protective Equipment (PPE), and the diversification of care pathways; and reducing all surgical activities by identifying the cohort of patients to be subjected to emergency or elective surgery.

The rapidly evolving situation allowed for only the acquisition of knowledge, mainly behavioral and epidemiological, to take place in real time. From this perspective, with the beginning of the national and international health crisis, every surgical department faced many changes and challenges. In our General Surgery Department in the Academic Hospital of Udine, FVG, north-eastern Italy, we adopted several empirical recommendations. These are summarized in Table 1.

**Table 1:** Empiric decisions taken in the first phase of the COVID 19 pandemic at our surgical department (Academic Hospital of Udine, FVG, North-Eastern Italy)

Working environment	Safe practices
Safety	
Staff (surgeons, nurses, support staff)	Always practice hand hygiene and social distancing and wear a surgical mask (compatible with the activities)
	Always use PPE when a patient may be COVID-19 positive or suspected
	Undertake COVID-19 testing if there is a risk of contact
	Watch educational videos such those demonstrating the ‘donning/doffing procedure’
	Arrange remote multidisciplinary meetings
Patients	COVID-19 tests should be done for all patients admitted to any surgical department, both in elective and emergency settings (when possible)
	Abdominal CT scans in the emergency setting should be extended to the chest, with an additional COVID 19 nasal swab test
	A COVID-19 test should be done before endoscopic procedures
Pathways	Suspension of elective outpatient activities
	Identification of separate care and diagnostic pathways for COVID-19 positive or suspect cases vs. COVID-19 negative cases
	Hospital visitors are banned
	Opt for remote contact with domiciled patients via telemedicine and telephone clinics
Indications for surgery	Elective surgery suspended for uncomplicated non urgent benign pathologies
	Oncological surgery
	Urgency/emergency surgery
	Organ transplant surgery
Operating room	Surgical rooms and dedicated ICU for positive or suspicious COVID-19 cases differentiated from COVID-19 negative cases
	The number of staff in the operating room should be kept to a minimum number, excluding all those who are not participating in the procedure
	During intubation and extubation, only personnel dedicated to the anaesthetic procedure should be present
	Mandatory wearing of FFP_2_/FFP_3_ masks for all procedures for the whole team
	Laparoscopic control of CO_2_ leakage through filtration systems

## Aims of this report

The first scientific contributions, national guidelines, and recommendations of good practice issued by scientific societies on the surgical activity at the time of COVID-19, are now available. The aim of this report is to review the available guidelines and recommendations for the management of surgical activities during the COVID-19 pandemic, analyzing and discussing the principle suggestions, concordance, and discordance.

## Method

**Fig. 1:**
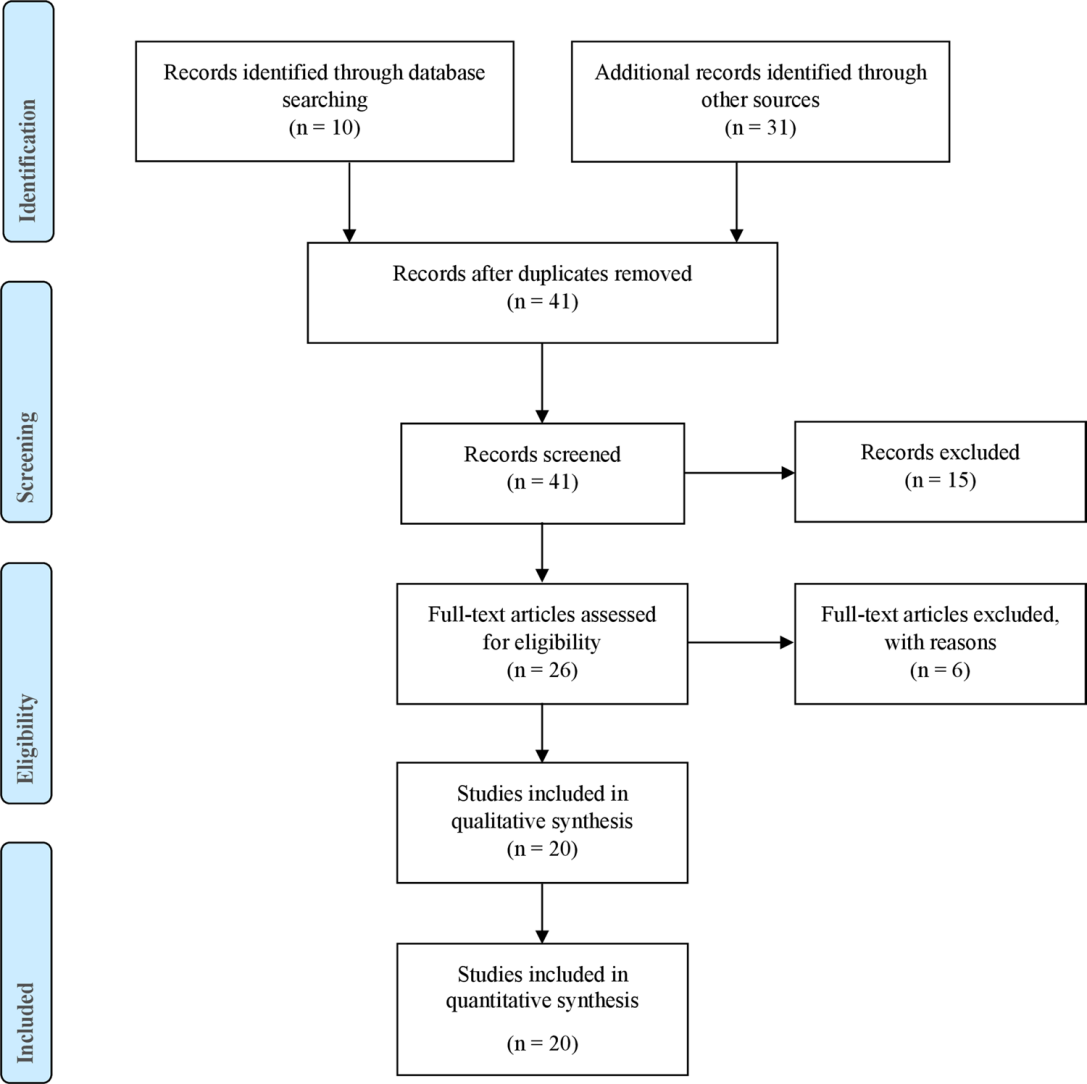
Study attrition diagram

The literature was reviewed by two independent reviewers (A.P. and C.B.) to identify all available reports. Selection criteria included guidelines, consensus recommendations, and scientific societies’ position statements on surgery and COVID-19. PubMed Database, Google, and Google Scholar were searched using the key words: “SARS-COV-2”, “COVID-19”, “SURGERY”, “RECOMMENDATIONS”, “GUIDELINE” and “TRIAGE”, both as single terms and in combination. The search was limited to publications in English and Italian and closed on May 6, 2020. All results were evaluated by the two reviewers. Any references that were not found initially were also considered. Reports were included if recommendations were relevant to the field of general surgery, with both general and specific suggestions about the indication for surgery, pre- and postoperative planning and operating theatre preparation. Studies were excluded if they were not relevant to the field of general surgery or if they described less than four of the five topics of interest (Table 2). Of the 41 initially identified articles, 20 met the inclusion criteria and 21 were found not to be relevant and were excluded. The data collected were entered into an Excel database to stratify the main “recommendations” in the individual studies and highlight recommendations common across publications. Of the 20 papers [9–28] included, 15 were national and international recommendations, guidelines, and general indications reported by scientific societies and 5 were scientific articles (Fig. 1).

**Table 2:** Retrieved suggestions and indications. (CT: computed tomography; NO: negative recommendation; OP: optional recommendation; UA: unavailable recommendation; YES: positive recommendation)

	Origin	International																Italian			
	Electronic Sources	PubMed—NCBI								Scholar		Google									
	Society/Journal	AOS1 [16]	AOS2 [27]	AJS [28]	BT [26]	CAS [15]	IJS [9]	JVS [10]	WJES [17]	ABC [20]	TJCD [14]	4open [11]	ACPGBI [22]	ACS [18]	ESSO [23]	RCS [19]	SAGES [13]	ACOI [25]	AICO [12]	SICE [24]	SICO (21)
General considerations	Non operative management	UA	UA	UA	YES	UA	YES	YES	UA	OP	UA	YES	OP	OP	YES	YES	UA	UA	UA	UA	OP
	Personal protective equipment (PPE)	YES	YES	YES	YES	YES	YES	YES	YES	YES	YES	YES	UA	YES	YES	YES	YES	YES	YES	YES	YES
Surgical indications	Elective surgery for benign pathologies	UA	NO	NO	NO	UA	NO	NO	UA	NO	NO	NO	NO	NO	NO	UA	NO	NO	UA	NO	NO
	Emergency surgery	YES	YES	YES	YES	UA	YES	YES	YES	YES	YES	YES	YES	YES	UA	YES	YES	YES	UA	YES	UA
	All surgical oncology	UA	NO	NO	NO	UA	NO	NO	YES	NO	UA	NO	NO	NO	NO	UA	NO	NO	UA	NO	NO
	Selected surgical oncology	UA	YES	UA	YES	UA	YES	YES	YES	OP	UA	YES	YES	YES	YES	UA	YES	YES	UA	YES	YES
Preoperative phase	Preoperative COVID 19 swab	UA	UA	YES	YES	UA	YES	UA	YES	UA	YES	YES	UA	UA	UA	YES	YES	YES	UA	YES	YES
	Preoperative chest CT scan	OP	UA	UA	YES	UA	NO	UA	YES	UA	UA	OP	UA	UA	UA	YES	YES	YES	UA	UA	NO
	Need of Multidisciplinary Team	UA	UA	UA	YES	UA	UA	YES	YES	UA	YES	YES	YES	UA	YES	UA	YES	UA	YES	YES	YES
Operating theatre	Dedicated room	YES	YES	YES	YES	YES	UA	YES	YES	YES	YES	YES	UA	YES	UA	UA	YES	YES	YES	YES	UA
	Limited / Dedicated personnel	UA	YES	YES	YES	YES	UA	UA	YES	YES	YES	YES	UA	YES	YES	YES	YES	YES	YES	YES	YES
	Specific protocol	YES	YES	UA	UA	YES	YES	UA	YES	YES	YES	YES	UA	UA	UA	UA	UA	YES	YES	YES	UA
	Anesthetic considerations	UA	UA	YES	YES	NO	UA	UA	YES	UA	UA	YES	UA	YES	UA	YES	UA	YES	YES	YES	UA
	Laparoscopic surgery	OP	UA	YES	YES	UA	NO	YES	YES	UA	OP	OP	OP	OP	UA	OP	OP	OP	UA	OP	OP
Postoperative phase	Specific indications for disinfection of operating rooms	YES	UA	UA	UA	YES	YES	UA	YES	YES	YES	YES	UA	UA	UA	UA	UA	UA	YES	YES	UA
	Postoperative indications	UA	YES	YES	YES	UA	YES	UA	YES	UA	YES	YES	UA	YES	UA	UA	YES	UA	YES	UA	UA

### General considerations: protection and surgical indications

The first fundamental aspect considered in the articles we reviewed is protection of the surgical team against COVID-19 when encountering positive or suspect cases, and the importance of correct use of personal protective equipment (PPE) such as surgical caps, protective masks (N95 or FFP_2_/FFP_3_), disposable overcoats, gloves, goggles or transparent barriers, and powered air-purifying respirators (PAPR). The latter is specifically recommended during aerosol generating procedures (AGPs), such as intubation and extubation, or operations on the respiratory tract [12–17]. The high rate of false negative tests (about 30%) has led us to consider and to treat as positive those patients with a negative test result, but who are either symptomatic or have radiological features of COVID-19 [29]. Moreover, asymptomatic COVID-19 infected patients are a known source of infection and may present with a negative test result. Therefore, PPE should be used for all surgical procedures [9–21,23–28], including for patients with negative test results [29]. Correct ‘donning and doffing’ training including simulation videos should be implemented [12].

The second important issue considered is to identify which surgical procedures are indicated and should proceed despite the added risks of surgery associated with the pandemic. Almost all the published articles agreed with the suspension of elective procedures for benign pathologies, except for complicated cases that need emergency/urgent treatment [9–11,13, 14,18, 20–28]. These precautions have been recommended to reduce the chance of overwhelming the hospital system and to reduce the risk of cross infection. The great majority of articles reviewed agree that emergency surgery must proceed [9–11, 13, 14, 16–20, 22, 24–28]. On the other hand, conservative treatment like antibiotics or radiological drainage should be considered if clinically possible for some situations that would usually warrant emergency surgery, such as uncomplicated acute appendicitis or acute cholecystitis [9–11, 18–23, 26]. In emergency bowel resection, stoma creation may be a better option than primary anastomosis to reduce the risk of complications and infections [19].

Most major recommendations indicate continuation of the multidisciplinary approach for surgical oncology patients. However, more conservative and non-invasive approaches such as neo-adjuvant chemotherapy and radiotherapy should be considered and where possible, endoscopic resection performed or interventions delayed for low-grade neoplasms [14, 18, 20–22]. These steps are recommended mainly when there are limited resources and they take into consideration the increased risk of perioperative morbidity and mortality [20]. The American College of Surgeon (ACS) and the Italian Society of Surgical Oncology (SICO) propose criteria for priority access to surgical treatments in relation to the degree of disease and type of tumor. The ACS also hypothesizes three phases of epidemic escalation with three scenarios of available resources associated with increasingly stringent surgical priority criteria [18,21].

Given the medical and legal implications that the above choices carry, there must be a decision-making phase to identify the most appropriate therapeutic plan for the patient, supported by a multidisciplinary team [13, 21–23]. The activation of telemedicine meetings is desirable [21]. Even for postoperative complications, a bridge to surgery should be created with alternative procedures whenever possible, for example, by stenting for colonic obstruction [21]. The risk of anastomotic leak should be minimized by such means as choosing Hartmann’s procedure at the expense of primary anastomosis in colorectal surgery [22]. For COVID-19 positive cancer patients, surgery should always be postponed until the swab is negative [21].

### Preoperative phase: hospitalization and screening

The recommended practices for admitting surgical patients to hospital for elective surgery involve redefining the pathways and implementing strict screening for COVID-19. Non-urgent outpatient clinical appointments should be cancelled, except when physical evaluation by the doctor is required. Telemedicine, telephone clinics, and other forms of remote patient management are preferred [13, 23, 24]. Protecting patients and stopping the spread of infection should be facilitated by establishing different pathways to separate suspect or confirmed COVID-19 patients from non-COVID-19 patients [17].

During the COVID-19 pandemic, all patients requiring surgery should be considered potentially infected. In this regard, the suggestion is to perform the screening test before admission to prevent viral spread and protect patients from possibly a worse postoperative outcome as a result of concurrent infection [13, 21, 29]. The role of chest CT scan for preoperative screening is less clear. The Italian Hospital Surgeons Association (ACOI) recommends a chest CT scan for all patients who undergo surgery for non-deferrable oncological pathology and for those who require emergency management in general [25]. In the emergency setting, it is recommended to perform an extended chest CT scan for those patients who need a CT of the abdomen or pelvis [19, 25]. However, the Italian Society of Oncological Surgery (SICO) emphasizes that because of the non-specificity of signs and patterns in COVID-19 pneumonia, a chest CT scan cannot be used to express a diagnostic judgment of certainty and should therefore be excluded from screening investigations [21].

When patients require emergency surgical treatment that cannot be postponed, in the absence of adequate screening, PPE and all precautions must be implemented to protect the healthcare team during surgery and peri-operatively [18]. It is pivotal to work with the multidisciplinary team to prioritize the patient's need for surgery in terms of possible hospitalization in an intensive care unit, risk of complications, the possible alternative of neo-adjuvant and adjuvant treatments, and available resources [18].

### Intraoperative phase: operating room, behavior, and operating technique

The need for different pathways for COVID-free and COVID-19 positive/suspect patients proved a major change in the immediate onset of an epidemic outbreak. For this reason, it is mandatory to manage COVID-19 positive patients separately, establishing in-house protocols and well-coded intra-hospital pathways [12,14–17, 20, 24, 25]. From this perspective, there must be dedicated operating rooms for COVID-positive or suspect patients, especially in emergency situations where screening cannot be done. The operating room used should ideally have negative pressure ventilation systems or at least high-pressure flows, and the health care teams must be specifically trained for emergency management and limited to the minimum number of team members necessary [12, 18, 26]. According to the WHO and Centers for Diseases Control and Prevention (CDC) recommendations, healthcare staff in operating theatres must always use PPE regardless of the COVID status of a patient [13, 16, 24]. A further criticism of the analyzed documents was the high risk of aerosol generation and therefore, of infection during anesthetic maneuvers. The prevailing suggestion is to intubate in the operating room, with minimum personnel present. All team members must use PPE [15]. Moreover, the surgical team should be kept to a minimum and with the highest level of expertise available [23, 26]. Some investigators recommend the suspension of surgical training for residents and students in the operating room [23].

It was also highly recommended to limit aerosol and smoke generation maneuvers during surgical procedures to prevent potential contamination. These statements include both open and laparoscopic surgery. Electrocautery should be chosen over energy devices such as radiofrequency and ultrasound. Direct aspiration of fumes in open surgery is also suggested. This includes the Luer-Lock valve and other specific filters for pneumoperitoneum ultrafiltration in laparoscopy. The use of filter masks such as FFP2 or N95, is indicated for personnel safety [13, 14, 16, 19,21, 24, 25].

The use of laparoscopic surgery is a debated topic and without unequivocal opinion as to whether it carries a higher risk of transmission of infection from the aerosolization of pathogens to health care personnel than open surgery. For this reason, the choice is left to individual surgical teams, evaluating risks and benefits such as shorter hospitalization and fewer complications on a case-by-case basis [13, 14, 16, 18, 19, 21, 24, 25]. When choosing the intervention, it is good practice to first consider the category with the lowest risk of complications for the same therapeutic outcome [19]. Other suggestions for laparoscopic surgery include smaller skin incisions to reduce possible gas leaks, the use of a low-pressure pneumoperitoneum, complete CO_2_ runoff before removal of surgical pieces and trocars, and avoidance of hand-assisted techniques [13, 24, 25].

### Postoperative phase: management aspects

Once surgery is completed, the management of patients and the organization of resources must be done carefully and not underestimated. PPE used by health care personnel must be disposed of correctly and the environment and equipment used during the operation, disinfected meticulously. This should be done by following specific checklists, particularly if the patient has suspect or confirmed COVID-19 positivity [12, 14, 17].

Postoperative patient monitoring must be carried out with the appropriate precautions in the operating room, especially extubation, when the patient is likely to cough. All postoperative procedures must be done with full protection such as negative pressure and PPE, including PAPR, and with the minimal number of staff needed [12, 19, 25]. Once the patient’s condition has stabilized, they can be transferred to the intensive care unit or ward, following specific pathways and limiting contact [14, 22]. The outcome of these recommendation is likely to increase the time between cases to allow for patient management and proper sanitization of the environment [12, 15]. Table 3 summarizes these practical recommendations.

**Table 3:** A summary of useful and practical recommendations regarding surgical response to COVID-19 crisis

Recommendations for safe practices	
General considerations: protection and surgical indications	
	PPE should be used for all surgical procedures
	Emergency surgical activity must be maintained
	Conservative treatment should be considered instead of surgery
Preoperative phase: hospitalization and screening	
	Non-urgent outpatient clinical appointments should be cancelled
	All patients requiring surgery should be considered potentially infected
Intraoperative phase: operating room, behaviour and operating technique	
	Identify dedicated operating theatres for patients with positive or suspected COVID-19
	Healthcare staff in operating theatres should always use PPE regardless of the patient’s COVID-19 status
	Perform intubation in the operating room, with only the minimum personnel necessary for the procedure
	Surgical team should be reduced to a minimum number
Postoperative phase: management aspects	
	Extubating and monitoring maneuvers should be carried out with full protection and with the minimal number of staff needed

## Comment

Approximately 1 month after the pandemic was declared, many of the surgical management recommendations were insufficient for all hospital environments and correlated with a low degree of evidence. Thus, a continuous updating of recommendations and guidelines is required according to the evolving scientific evidence that is continually changing during this pandemic [30]. We analyzed all the published data on this subject to maximize the safety of patients, surgeons, and other healthcare professionals during the SARS-CoV-2 pandemic. The recommendations outlined in this brief review are good practices that require scientific validation in most cases, and whose applications must be contextualized to local situations, individual hospitals, positive COVID-19 patient load, and availability of resources.
